# Autologous skull graft resorption following cranioplasty: a case report and literature review

**DOI:** 10.3389/fsurg.2026.1838348

**Published:** 2026-06-24

**Authors:** Miao Yuan, Huarong Wang, Yurong Wang

**Affiliations:** 1 Department of Neurosurgery, Sichuan Mianyang 404 Hospital·Affiliated Hospital of Southwest University of Science and Technology, Mianyang, China; 2 Department of Neurosurgery, Jiange County People’s Hospital, Guangyuan, China; 3 Department of General Medicine, Mianyang Hospital Affiliated to University of Electronic Science and Technology·Mianyang Central Hospital, Mianyang, China

**Keywords:** autologous cranioplasty, autologous skull graft resorption, cerebral vascular malformation, decompressive craniectomy, ventriculoperitoneal shunt

## Abstract

**Background:**

Autologous cranioplasty remains the first-line option for skull defect repair after decompressive craniectomy, with well-documented advantages of excellent biocompatibility, no immune reaction, and favorable cosmetic outcomes. Autologous skull graft resorption (ASGR) is a common postoperative complication that may lead to recurrent defects, poor cosmesis, and even neurological compromise. Patients with prior ventriculoperitoneal (VP) shunt are at substantially higher risk, yet long-term follow-up data and optimal management strategies in this subgroup remain poorly defined.

**Case description:**

A 27-year-old male presented with spontaneous intracerebral hemorrhage secondary to cerebral arteriovenous malformation. He underwent emergency hematoma evacuation, AVM resection, decompressive craniectomy, and ePTFE duraplasty. Postoperatively, VP shunt was placed for communicating hydrocephalus. Autologous cranioplasty was performed two months later. Serial cranial CT follow-up revealed progressive ASGR starting at six months, with marked regional predominance in the occipital stress-bearing area. One year postoperatively, shunt pressure was adjusted upward for decompression window depression. The patient remained asymptomatic throughout a 3-year follow-up without revision cranioplasty.

**Conclusion:**

ASGR in VP-shunted patients is driven primarily by chronic intracranial hypotension. This case demonstrates that long-term conservative surveillance with individualized shunt pressure titration is safe and effective for asymptomatic moderate-to-severe ASGR, and that timely pressure adjustment may significantly slow resorption progression. Regional mechanical stress further exacerbates resorption. Routine imaging and close intracranial pressure monitoring are essential for high-risk patients.

## Introduction

Cerebral vascular malformations are a major cause of spontaneous intracerebral hemorrhage in young adults, associated with high morbidity and mortality (1). Decompressive craniectomy is a life-saving emergency procedure for severe hemorrhage, but it leaves large skull defects that impair cranial protection, cerebral hemodynamics, and cosmesis (2). Cranioplasty is therefore a mandatory reconstructive step to restore cranial integrity and physiological function.

Autologous bone graft is the gold standard for cranioplasty due to its biological compatibility and capacity for osseointegration (3). However, autologous skull graft resorption (ASGR) remains a frequent complication, with reported rates of 5%–30% (4). Multiple risk factors have been identified, including patient age, nutritional status, surgical technique, graft preservation, and postoperative infection (5).

Patients requiring VP shunt after craniectomy represent a distinct high-risk group for ASGR (6). Chronic intracranial hypotension and cerebrospinal fluid circulatory disturbance are considered key mechanisms. Although VP shunt as a risk factor is well recognized, long-term natural history, regional resorption patterns, and the efficacy of conservative management remain underreported. Moreover, the impact of shunt pressure adjustment on ASGR progression has not been fully elucidated (7).

This report describes a 3-year follow-up of ASGR in a young patient with prior VP shunt. Serial CT imaging was used to quantify resorption and identify regional patterns. We also discuss the underlying mechanisms, preventive strategies, and individualized management, with emphasis on the role of shunt pressure optimization in asymptomatic patients (8).

## Case description

**Figure 1:**
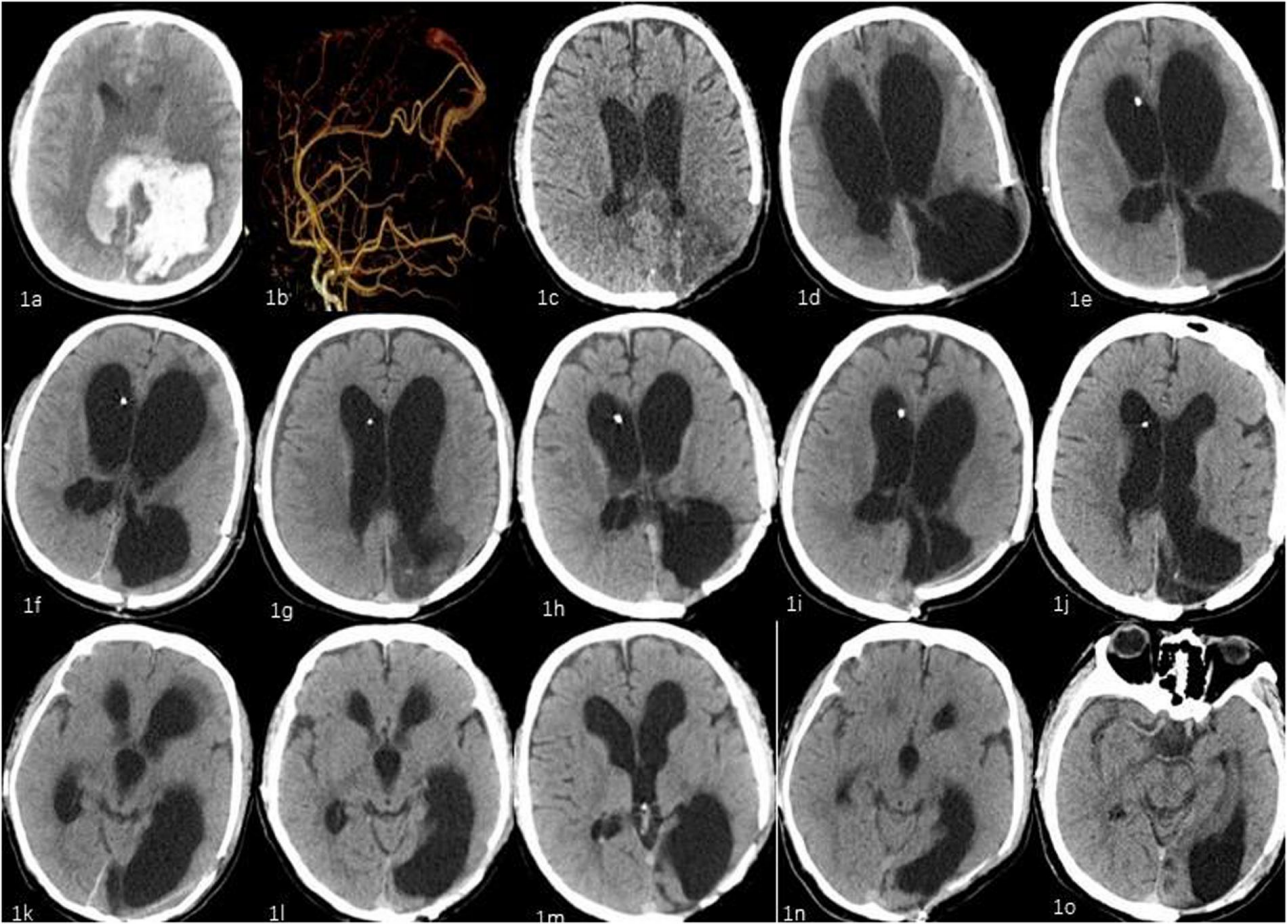
Serial imaging findings throughout the patient's clinical course **(a)** axial cranial CT demonstrating a large hyperdense hematoma involving the splenium of the corpus callosum and left parieto-occipital lobe.**(b)** Axial cranial CTA revealing a left parieto-occipital arteriovenous malformation with dilated feeding arteries and tortuous draining veins.**(c)** Postoperative axial CT following emergency craniotomy for hematoma evacuation, AVM resection, and decompressive craniectomy.**(d)** Axial CT showing communicating hydrocephalus at 1 month after the index surgery.**(e)** Postoperative axial CT after VP shunt placement demonstrating resolution of hydrocephalus.**(f,k)** Postoperative axial and coronal CT images at 1 month post-VP shunt placement showing autologous cranioplasty for the left parieto-occipital skull defect.**(g,l)** Axial and coronal CT images at 3 months post-cranioplasty showing stable bone flap position with no resorption.**(h,m)** Axial and coronal CT images at 6 months post-cranioplasty showing mild focal resorption and thinning of the bone flap.**(i,n)** Axial and coronal CT images at 1 year post-cranioplasty showing significant bone resorption with focal loosening and decompression window depression.**(j,o)** Axial and coronal CT images at 3 years post-cranioplasty showing progressive bone resorption with regional variability. All images are submitted as a 3 × 5 grid at 300 DPI resolution with enlarged subfigure labels **(a–o)** for optimal readability.

A 27-year-old male, non-smoker, non-drinker, with no prior medical or family history, presented with sudden loss of consciousness and generalized seizures [GCS 6; left pupil dilated, nuchal rigidity, bilateral decerebrate posturing]. Emergency cranial CT showed a large hematoma in the splenium of the corpus callosum and left parieto-occipital lobe (Figure 1a). CTA confirmed a left parieto-occipital arteriovenous malformation (Figure 1b).

Emergency craniotomy was performed for hematoma evacuation, AVM resection, and decompressive craniectomy (12  ×  10 cm bone flap). ePTFE duraplasty was performed for watertight closure without dural tenting sutures (Figure 1c). The bone flap was rinsed, wrapped in sterile gauze, and stored at −80 °C. Postoperatively, the patient recovered with residual lower extremity weakness (grade 2/5). One month later, he developed headache, nausea, and vomiting. CT confirmed communicating hydrocephalus (Figure 1d). A programmable VP shunt (Codman Hakim) was inserted with an initial pressure of 80 mmH₂O, achieving rapid symptom resolution (Figure 1e).

Two months after shunt placement, autologous cranioplasty was performed. The cryopreserved bone flap was thawed in 37 °C saline for 30 min and fixed with six titanium plates (Figures 1f–k). Postoperative recovery was uneventful, and the patient was discharged on postoperative day 10 with improved lower extremity strength (grade 3/5).

### Follow-up and imaging

3 months: No resorption; hydrocephalus controlled; strength improved to grade 4/5 (Figures 1g–l).

6 months: Mild focal resorption (12.3%); asymptomatic (Figures 1h–m).

1 year: Significant resorption (35.6%) with decompression window depression. Shunt pressure increased to 120 mmH₂O, with complete resolution of depression within two weeks (Figures 1i–n).

3 years: Progressive resorption (58.2%) with marked regional predominance in the occipital area (72.4% vs. 31.8% in parietal region) (Figures 1j–o).

The residual bone flap covered approximately 85% of the original defect (18.2 cm^2^/21.4 cm^2^). Throughout follow-up, the patient remained neurologically stable without infection, hemorrhage, or seizures. At one year, the risks of revision surgery were thoroughly discussed. The patient expressed strong preference for conservative care due to concern for operative complications and satisfaction with functional and cosmetic outcomes. Long-term surveillance was adopted (Table 1).

**Table 1:** Clinical timeline.

Time Point	Clinical Event	Intervention	Outcome
Day 0	ICH, AVM, herniation	Emergency craniotomy + duraplasty	GCS 13 post-op
1 month	Hydrocephalus	VP shunt (80 mmH₂O)	Symptoms resolved
2 months	Skull defect	Autologous cranioplasty	Uneventful recovery
3 months	Routine follow-up	None	No resorption
6 months	Mild ASGR	None	Asymptomatic
1 year	Moderate ASGR + depression	Shunt pressure ↑ to 120 mmH₂O	Depression resolved
3 years	Severe regional ASGR	Conservative follow-up	Neurologically stable

## Discussion

ASGR in VP-shunted patients is multifactorial, driven primarily by chronic intracranial hypotension, local perfusion impairment, and mechanical stress (9). This case demonstrates that asymptomatic patients with moderate-to-severe ASGR can be safely managed conservatively with individualized ICP control, and that shunt pressure titration may slow progression.

### Intracranial hypotension as the core mechanism

VP shunt-induced chronic hypotension reduces brain bulk and diminishes contact between the bone flap and underlying dura, impairing revascularization and osteogenesis (10). Hypotension suppresses osteoblast activity and enhances osteoclast function, accelerating resorption (11, 12). In this case, increasing shunt pressure from 80 to 120 mmH₂O resolved depression and reduced annual resorption from 35.6% to 11.3%, confirming the critical role of ICP normalization (13). While our findings support the benefit of shunt pressure adjustment, it should be noted that some smaller studies have not observed a significant reduction in ASGR progression with this intervention, highlighting the need for larger prospective trials to confirm its efficacy.

### Regional mechanical stress

Resorption was significantly worse in the occipital region, consistent with biomechanical loading during posture and movement (14). Stress-induced osteoclast activation disrupts bone remodeling (15). This regional pattern should inform material selection and follow-up protocols, with closer surveillance of stress-bearing areas.

### Duraplasty and graft preservation

ePTFE duraplasty may increase ASGR risk by impairing osseointegration. A 2025 systematic review confirmed that synthetic dural substitutes are associated with a 2.3-fold higher risk of ASGR compared to autologous dura (16, 17). Our institutional retrospective analysis of 327 consecutive autologous cranioplasty cases (2021–2025) further supports this finding, with resorption rates of 28.7% with ePTFE vs. 11.2% with autologous temporalis fascia (*P* < 0.01). Cryopreservation at −80 °C also reduces osteoblast viability, with a prospective study showing a 30% decrease in osteoblast activity after 3 months of storage (18). Additionally, the 2–3 mm shrinkage of the cryopreserved bone flap created a narrow gap between the flap and the skull margin, which further reduced the contact area for revascularization and may have contributed to the accelerated resorption observed in this case.

### Management and novel insights

Most studies focus on risk factors; few report long-term conservative outcomes or pressure titration effects (19). This case provides three key clinical findings:

1. Asymptomatic moderate-severe ASGR is manageable conservatively (20).
2. Regional resorption patterns guide risk stratification (21).
3. Shunt pressure titration can slow ASGR progression (22).

### Cranioplasty materials

Autologous bone remains first-line; titanium/PEEK are alternatives for large defects (23). Hydroxyapatite (HA) is osteoconductive but brittle and infection-prone, unsuitable for stress areas or VP-shunted patients (24, 25). In this specific case, HA would not have been a suitable alternative due to the large defect size (12 × 10 cm) and the occipital location, which is subject to high mechanical stress during daily activities.

### Achievement of cranioplasty objectives

Cranioplasty serves three fundamental purposes: protection of intracranial contents, normalization of intracranial pressure, and aesthetic restoration. In this case, these objectives were partially achieved:

1. Intracranial protection: The residual bone flap covered 85% of the original defect (18.2 cm^2^), below the 25 cm^2^ threshold for clinically significant functional impairment. No traumatic brain injury occurred during 3-year follow-up, but the patient was advised to avoid contact sports.
2. Intracranial pressure normalization: Fully achieved after shunt pressure adjustment to 120 mmH₂O, with resolution of decompression window depression and stable cerebral perfusion.
3. Aesthetic restoration: Mild depression at the defect site was present, but the patient reported satisfaction and declined revision surgery for cosmetic reasons alone.

### Strengths and limitations

Strengths: 3-year quantitative follow-up, regional pattern description, and demonstration of shunt pressure titration effect. Limitations: single case, no histology, single preservation technique, limited neuropsychological data. Additionally, as a single-center experience, our findings may not be fully generalizable to diverse patient populations or different healthcare settings (26).

### Clinical implications

For VP-shunted patients undergoing autologous cranioplasty:

1. Maintain ICP >100 mmH₂O to reduce resorption risk, as recommended by Servadei et al. (2015) and supported by our clinical experience (27, 28).
2. Use autologous dura when possible to promote osseointegration.
3. Perform long-term CT surveillance, with particular attention to stress-bearing regions.
4. Adopt conservative management for asymptomatic ASGR, with shared decision-making between clinicians and patients.

## Conclusion

ASGR after autologous cranioplasty in VP-shunted patients is driven by chronic intracranial hypotension, regional mechanical stress, and surgical factors. Asymptomatic moderate-to-severe ASGR can be safely managed with conservative surveillance and individualized shunt pressure titration, which may slow progression. Long-term imaging and ICP monitoring are essential for high-risk patients. This case supports personalized management and provides practical guidance for clinical practice.

## Data Availability

The original contributions presented in the study are included in the article/Supplementary Material, further inquiries can be directed to the corresponding author.

## References

[1] He Q Huo R Sun Y Zheng Z Xu H Zhao S. Cerebral vascular malformations: pathogenesis and therapy. Med Comm. (2024) 5:e70027. 10.1002/mco2.70027 PMC11625509 39654683

[2] Wang K Guo H Zhu Y Li J Niu H Wang Y. Improved strategy for post-traumatic hydrocephalus following decompressive craniectomy: experience of a single center. Front Surg. (2023) 9:935171. 10.3389/fsurg.2022.935171 36684286 PMC9852628

[3] Yuruk B Tekiner A Erdem Y Celik H Yildirim ME Kurtulus A. Factors affecting resorption after autologous cranioplasty. Turk Neurosurg. (2024) 34:600–6. 10.5137/1019-5149.JTN.44249-23.2 38874238

[4] Snee I Gensler R Dowlati E Parikh RP Felbaum D. Autologous vs synthetic cranioplasty. Acta Neurochir (Wien). (2025) 167:58. 10.1007/s00701-025-06480-0 40035779 PMC11880053

[5] Yang J Sun T Yuan Y Li X Zhou Y Guan J. Risk factors for bone flap resorption after autologous bone cranioplasty: protocol for a systematic review and meta-analysis. Medicine (Baltimore). (2020) 99:e21035. 10.1097/MD.0000000000021035 32664110 PMC7360233

[6] Ritter L Strohhäcker K Schebesch KM Eibl T Höhne J Liebert A. Complications after autologous cranioplasty following decompressive craniectomy. Acta Neurochir (Wien). (2024) 166:380. 10.1007/s00701-024-06282-w 39320557 PMC11424706

[7] Fan MC Wang QL Sun P Zhan S-H Guo P Deng W-S. Cryopreservation of autologous cranial bone flaps for cranioplasty: a large sample retrospective study. World Neurosurg. (2018) 109:e853–9. 10.1016/j.wneu.2017.10.112 29107719

[8] Signorelli F Giordano M Caccavella VM Ioannoni E Gelormini C Caricato A. A systematic review and meta-analysis of factors involved in bone flap resorption after decompressive craniectomy. Neurosurg Rev. (2022) 45:1915–22. 10.1007/s10143-022-01737-z 35061139

[9] Ernst G Qeadan F Carlson AP. Subcutaneous bone flap storage after emergency craniectomy: cost-effectiveness and rate of resorption. J Neurosurg. (2018) 129:1604–10. 10.3171/2017.6.JNS17943 29303450

[10] Birgersson U Wettervik TS Sundblom J Linder LKB. The role of autologous bone in cranioplasty. A systematic review of complications and risk factors by using stored bone. Acta Neurochir (Wien). (2024) 166:438. 10.1007/s00701-024-06312-7 39495337

[11] Lee SH Yoo CJ Lee U Park CW Lee SG Kim WK. Resorption of autogenous bone graft in cranioplasty: resorption and reintegration failure. Korean J Neurotrauma. (2014) 10:10–4. 10.13004/kjnt.2014.10.1.10 27169026 PMC4852591

[12] Göttsche J Mende KC Schram A Westphal M Amling M Regelsberger J. Cranial bone flap resorption-pathological features and their implications for clinical treatment. Neurosurg Rev. (2021) 44(4):2253–60. 10.1007/s10143-020-01417-w 33047218 PMC8338853

[13] Chan DYC Mok YT Lam PK Tong CSW Ng SCP Sun TFD. Cryostored autologous skull bone for cranioplasty? A study on cranial bone flaps’ viability and microbial contamination after deep-frozen storage at −80 °C. J Clin Neurosci. (2017) 42:81–3. 10.1016/j.jocn.2017.04.016 28431953

[14] Dinc N Won SY Brawanski N Eibach M Quick-Weller J Konczalla J. Differences in bleeding patterns and outcome after intracerebral hemorrhage due to vascular malformations. PLoS One. (2019) 14:e0217017. 10.1371/journal.pone.0217017 31120937 PMC6532871

[15] Punchak M Chung LK Lagman C Bui TT Lazareff J Rezzadeh K. Outcomes following polyetheretherketone (PEEK) cranioplasty: systematic review and meta-analysis. J Clin Neurosci. (2017) 41:30–5. 10.1016/j.jocn.2017.03.028 28377284

[16] Chasles OG Kokot K Fercho J Siemiński M Szmuda T. Comparison of complications in early and late cranioplasty following decompressive craniectomy due to traumatic brain injury: systematic review and meta-analysis. J Clin Med. (2025) 14(12):4176. 10.3390/jcm14124176 40565921 PMC12194293

[17] Chibbaro S Zaed I Dannhoff G Todeschi J Mallereau CH Prisco L. Cranioplasty complications in severe traumatic brain injury: implications of timing of surgery, implant material and incidence of vetriculomegaly versus post-traumatic hydrocephalus. Neurosurg Rev. (2025) 48(1):659. 10.1007/s10143-025-03832-3 40974389

[18] Gerstl JVE Rendon LF Burke SM Doucette J Mekary RA Smith TR. Complications and cosmetic outcomes of materials used in cranioplasty following decompressive craniectomy-a systematic review, pairwise meta-analysis, and network meta-analysis. Acta Neurochir (Wien). (2022) 164(12):3075–90. 10.1007/s00701-022-05251-5 35593924

[19] Kim JH Kim JH Kwon TH Chong K Hwang SY Yoon WK. Aseptic bone flap resorption after cranioplasty with autologous bone: incidence, risk factors, and clinical implications. World Neurosurg. (2018) 115:e111–8. 10.1016/j.wneu.2018.03.197 29626687

[20] Alkhaibary A Alharbi A Alnefaie N Oqalaa Almubarak A Aloraidi A Khairy S. Cranioplasty: a comprehensive review of the history, materials, surgical aspects, and complications. World Neurosurg. (2020) 139:445–52. 10.1016/j.wneu.2020.04.211 32387405

[21] Joo JK Choi JI Kim CH Lee HK Moon JG Cho TG. Initial dead space and multiplicity of bone flap as strong risk factors for bone flap resorption after cranioplasty for traumatic brain injury. Korean J Neurotrauma. (2018) 14(2):105–11. 10.13004/kjnt.2018.14.2.105 30402427 PMC6218344

[22] Lee JH Chough CK Choi HJ Ko JK Cho WH Cha SH. Bone flap changes after cranioplasty using frozen autologous bone flaps: a three-dimensional volumetric reconstruction study. Yonsei Med J. (2019) 60(11):1067–73. 10.3349/ymj.2019.60.11.1067 31637889 PMC6813147

[23] Zanotti B Zingaretti N Verlicchi A Robiony M Alfieri A Parodi PC. Cranioplasty: review of materials. J Craniofac Surg. (2016) 27(8):2061–72. 10.1097/SCS.0000000000003025 28005754

[24] Carbonaro R Ghiringhelli G Nataloni A Amendola F Catapano S Vaienti L. Long-Term series of custom-bone hydroxyapatite cranioplasty: outcomes and survival at 15 years. J Craniofac Surg. (2025) 36(4):1263–6. 10.1097/SCS.0000000000011061 39953654

[25] Morselli C Zaed I Tropeano MP Cataletti G Iaccarino C Rossini Z. Comparison between the different types of heterologous materials used in cranioplasty: a systematic review of the literature. J Neurosurg Sci. (2019) 63(6):723–36. 10.23736/S0390-5616.19.04779-9 31599560

[26] Korhonen TK Salokorpi N Niinimäki J Serlo W Lehenkari P Tetri S. Quantitative and qualitative analysis of bone flap resorption in patients undergoing cranioplasty after decompressive craniectomy. J Neurosurg. (2019) 130(1):312–21. 10.3171/2017.8.JNS171857 29473777

[27] Honeybul S Ho KM. Decompressive craniectomy for severe traumatic brain injury: the relationship between surgical complications and the prediction of an unfavourable outcome. Injury. (2014) 45(9):1332–9. 10.1016/j.injury.2014.03.007 24704150

[28] Servadei F Iaccarino C. The therapeutic cranioplasty still needs an ideal material and surgical timing. World Neurosurg. (2015) 83(2):133–5. 10.1016/j.wneu.2014.08.031 25153284

